# Antioxidant-Rich Extracts of *Terminalia ferdinandiana* Interfere with Estimation of Cell Viability

**DOI:** 10.3390/antiox8060191

**Published:** 2019-06-22

**Authors:** Saleha Akter, Rama Addepalli, Michael E. Netzel, Ujang Tinggi, Mary T. Fletcher, Yasmina Sultanbawa, Simone A. Osborne

**Affiliations:** 1 Queensland Alliance for Agriculture and Food Innovation (QAAFI), The University of Queensland, Health and Food Sciences Precinct, 39 Kessels Rd, Coopers Plains, QLD 4108, Australia; saleha.akter@uq.edu.au (S.A.); m.netzel@uq.edu.au (M.E.N.); mary.fletcher@uq.edu.au (M.T.F.); y.sultanbawa@uq.edu.au (Y.S.); 2 CSIRO Agriculture and Food, 306 Carmody Road, St Lucia, QLD 4067, Australia; Rama.Addepalli@csiro.au; 3 Queensland Health Forensic and Scientific Services, Health and Food Sciences Precinct, 39 Kessels Rd, Coopers Plains, Qld 4108, Australia; ujang.tinggi@health.qld.gov.au

**Keywords:** *Terminalia ferdinandiana*, cell viability, MTS, phenolics, interference, antioxidants

## Abstract

The impact of plant extracts and phytochemicals on in vitro cell viability is usually assessed by employing cell viability assays dependent upon the activity of dehydrogenase enzymes. The CellTiter 96^®^ AQ_ueous_ One Solution Cell Proliferation Assay (CellTiter) was used to measure cell viability in response to antioxidant-rich extracts of *Terminalia ferdinandiana* fruits. Conflicting results were obtained from this assay whereby higher concentrations of extracts significantly increased cell viability compared to lower concentrations. Intrinsic reductive potential was observed in a cell-free system when extracts were added directly to the CellTiter assay reagent. To confirm this effect in a similar cell proliferation assay, we employed the CellTiter-Blue^®^ Cell Viability Assay and again observed increased viability with increased concentrations of the extracts and direct reduction of the assay reagent by the extracts in cell-free systems. In the search for a cell proliferation assay that would not be directly affected by the plant extracts, we identified the CyQUANT^®^ NF Cell Proliferation Assay that is based on the estimation of DNA content in viable cells. Cell viability decreased with increasing concentrations of the extracts. Accordingly, the results of the present study indicated that cell viability assays reliant upon dehydrogenase activity may lead to false positive results when testing antioxidant-rich plant extracts with intrinsic reductive potential, and alternative cell viability assays should be used to measure the cell viability.

## 1. Introduction

In vitro cell models are often employed as high throughput screening tools used to gather data sets that are difficult to obtain in vivo. For example, cell proliferation assays can be used to indicate cytotoxicity and inform animal and human studies targeted to assessing the biological function of new therapeutics or functional ingredients from fruits, vegetables, and other plant sources. Cell proliferation assays exploit various cell functions, such as mitochondrial enzyme activity, cell membrane permeability, ATP production, or measurements of cellular DNA content. The CellTiter 96^®^ AQueous One Solution Cell Proliferation Assay (Promega Corporation, Madison, WI, USA) is one such assay and contains a tetrazolium compound, MTS (3-(4,5-dimethylthiazol-2-yl)-5-(3-carboxymethoxyphenyl)-2-(4-sulfophenyl)-2H-tetrazolium inner salt), and an electron coupling reagent PES (phenazine ethosulfate). The MTS reagent is reduced by cells into a coloured 1-(4,5-dimethylthiazol-2-yl)-3,5-diphenylformazan product that is soluble in cell culture medium and measured by a change in absorbance at 490 nm. This reduction occurs in the presence of NADPH or NADH produced by dehydrogenase enzymes in metabolically active cells [1].

Studies have revealed that some compounds found in plant extracts interfere with the estimation of cell viability using related cell proliferation assays, like the MTT (3-(4,5-dimethylthiazol-2-yl)-2,5-diphenyltetrazolium bromide) assay, potentially leading to an overestimation of viable cells [2]. The MTT assay is based on the reduction of the tetrazolium dye MTT to insoluble formazan, while related tetrazolium dyes, such as MTS (3-(4,5-dimethylthiazol-2-yl)-5-(3-carboxymethoxyphenyl)-2-(4-sulfonyl)-2H-tetrazolium), are used in conjunction with the intermediate electron acceptor PMS (1-methoxy phenazine methosulfate). Some phenolics, like kaempferol and epigallocatechin-3-gallate found in green tea, have been reported to reduce MTT in cell-free media, and when in the presence of cells, interference of the phenolics with MTT can be reduced by washing the cells prior to adding the MTT reagent [3]. Other biological molecules, like proteins and carbohydrates with reductive capacity, can also reduce MTT-based reagents.

*Terminalia ferdinandiana* is a native Australian fruit that has been used by Indigenous people for centuries on hunting trips for quick energy and refreshment. Recent research has shown that *T. ferdinandiana* has the highest reported vitamin C content of all fruits, and is rich in other hydrophilic (phenolics, flavonols) and lipophilic (α-tocopherol, γ-tocopherol, δ-tocopherol, lutein, chlorophyll a and b) antioxidants, nutrients, and other phenolics such as ellagic acid [4,5]. Overall, ellagic acid and vitamin C are the most abundant, and possibly the most bioactive antioxidants present in *T. ferdinandiana* fruits [6]. To investigate the general safety and bioactivity of fruit extracts using various cell-based in vitro assays (like antioxidant or anti-inflammatory assays), initial assessments of cytotoxicity are needed to determine cell treatment ranges and ensure that bioactivity can be observed in viable cells. To identify the most suitable cell viability assay, different assays were used including the CellTiter 96^®^ AQ_ueous_ One Solution Cell Proliferation Assay, CellTiter-Blue^®^ Cell Viability Assay (Promega Corporation, Madison, WI, USA), and the CyQUANT^®^ NF Assay (Invitrogen™, Molecular Probes™, Thermo Fisher Scientific Corporation, Carlsbad, CA, USA). Based on the results observed in this study, the variable composition of different plant extracts necessitates careful selection of cell viability assays to remove, or at least reduce, direct interaction of extracts with cell viability reagents masking the actual impact of extracts on cell proliferation in vitro.

## 2. Materials and Methods

### 2.1. Preparation of T. ferdinandiana Extracts

#### 2.1.1. Plant Materials

Fully ripe and mature fruits of *T. ferdinandiana* were collected from the Northern Territory, Australia. A voucher specimen, AQ522453, was deposited at the Queensland Herbarium. The collected fruits were processed by Sunshine Tropical Fruit Products, Nambour, Queensland, Australia, to provide a seedless puree which was kept at −20 °C until further analysis. The puree was then freeze- dried and milled to powder. The milled freeze-dried powder was used throughout the study.

#### 2.1.2. Accelerated Solvent Extraction (ASE)

Accelerated solvent extraction (ASE) (Dionex ASE 200 system, Dionex Corp., Sunnyvale, CA, USA) was performed to prepare the extracts [7]. Briefly, a 10 mL stainless steel extraction cell was assembled and fitted with a 27 mm cell filter at the bottom end. Next, 1.0 g of freeze-dried powders of fruits of *T. ferdinandiana* was mixed with diatomaceous earth (DE) and placed in the cells. Methanol and water were used as the solvent. The ASE system parameters were as follows: temperature 60 °C for methanol, and 75 °C for distilled water; preheat 5 min; static time 5 min, eight extraction cycles, rinse volume 25% with fresh extraction solvent and purged with 150 psi for 60 sec. The cells containing the samples were prefilled with respective extraction solvent, pressurized, and heated with the extracts collected into 60 mL amber glass vials. To concentrate and dry the collected extracts, a miVac sample concentrator (GeneVac Inc., Gardiner, NY, USA) was used. The temperature for the evaporation of methanol was set at 50 °C and for water the temperature was set at 70 °C. After weighing, the concentrated extracts were stored at −20 °C for further analysis.

### 2.2. Cell Viability Assays

#### 2.2.1. Cell Culture Materials

Dulbecco’s modified eagle medium (DMEM), fetal bovine serum (FBS), penicillin and streptomycin, GlutaMAX, Hank’s Balanced Salt Solution (HBSS), Dulbecco’s phosphate buffered saline without calcium and magnesium (PBS), trypsin- Ethylenediaminetetraacetic acid (EDTA), trypan blue exclusion dye and non-essential amino acids (NEAA) were purchased from Invitrogen (Sydney, NSW, Australia). Nunc cell culture flasks and 96-well plates were from Sigma-Aldrich (Castle Hill, NSW, Australia). HT29-MTX-E12 cell lines and all other chemicals were purchased from Sigma-Aldrich (Castle Hill, NSW, Australia) unless otherwise specified.

#### 2.2.2. Routine Cell Culture

HT29-MTX-E12 cells were maintained in DMEM containing 0.1% MEM non-essential amino acid, 100 U/mL penicillin, 100 μg/mL streptomycin and 2 mM GlutaMAX supplemented with 10% FBS (*v*/*v*). Cells were cultured at 37 °C in 5% CO_2_ and 95% air. Cells were passaged at 80–90% confluency with media changed every 2–3 days. Subculturing was performed by washing the cells with PBS. Cell detachment from the vented culture flasks was done by adding 0.25% (*v*/*v*) trypsin-EDTA and the flasks were incubated for 1–2 min or until the cells were completely detached. Growth media was used to wash off the cells. Centrifugation at 1500× *g* was performed and old media was discarded. Fresh media was added to resuspend the cells. The resuspended cells were then used to further culture cells. HT29-MTX-E12 cells were used at a density of 4 × 10^4^ cells/well to perform the cell viability assays.

#### 2.2.3. In Vitro Cell Viability Assays

CellTiter 96^®^ AQ_ueous_ One Solution Cell Proliferation Assay was used to measure the effect of antioxidant-rich *T. ferdinandiana* extracts on the viability of human HT29-MTX-E12 cells. The cells (4 × 10^4^ cells/well) were grown in 96-well plates 24 h prior to treatment, producing 90% cell confluence. Cells were washed by replacing culture media with HBSS (100 μL/well) for 2 h. HBSS was removed and the cells were treated with methanolic and water extracts of fruits diluted in HBSS to produce a concentration range of 1000–10,000 µg/mL applied at 50 μL/well. The concentration range was selected to represent nontoxic, moderately, and highly cytotoxic treatments to establish a treatment response curve. Each treatment was applied for 2 h to mimic intestinal transit time and was compared with cell viability in response to HBSS only. After 2h, all treatments were removed and the cells were washed once with HBSS (74 μL/well). To measure cell viability, HBSS (100 μL/well) and CellTiter 96^®^ AQ_ueous_ One Solution Cell Proliferation Assay reagent (20 μL/well) were added to the washed cells and the cells were incubated for 120 min at 37 °C before absorbance was measured at 490 nm. An interference experiment was performed by adding 5 µL extracts to 95 µL HBSS in a 96-well plate using the same range of treatment concentrations. After 2 h incubation, 20 µL of CellTiter 96^®^ AQ_ueous_ One Solution Cell Proliferation Assay reagent were added to each well and cells were then incubated for 120 min at 37 °C before absorbance was measured at 490 nm using a Spectramax M3 multi-mode microplate reader (Molecular Devices, LLC, San Jose, CA, USA).

CellTiter-Blue^®^ Cell Viability Assay (Promega Corporation, Madison, WI, USA) was used to perform the interference experiment in a cell-free system. Briefly, 5 µL extracts were added to the 96-well plates to 95 µL HBSS using the same range of treatment concentrations used in the CellTiter 96^®^ AQ_ueous_ One Solution Cell Proliferation Assay. The plates were then shaken for 10 s. After 2 h incubation, 20 µL CellTiter-Blue^®^ Cell Viability Assay reagent was added to each well and the cells were then incubated for 120 min at 37 °C. Plates were again shaken for 10 s and fluorescences were recorded at 560/590 nm using Spectramax M3 multi-mode microplate reader.

CyQUANT^®^ NF Cell Proliferation Assay Kit is based on the measurement of cellular DNA content via fluorescent dye binding. The CyQUANT^®^ NF assay was employed against the extracts in a cell-free system as well as against HT29-MTX-E12 cells. Cell-free experiments were performed as per the method described in the CellTiter 96^®^ AQ_ueous_ One Solution Cell Proliferation Assay method section. The HT29-MTX-E12 cells (4 × 10^4^ cells/well) were grown in 96-well plates 24 h prior to the treatment. Cells were washed by replacing culture media with HBSS (100 μL/well) and cells were incubated for 2 h. HBSS was removed and the cells were treated with methanolic and water extracts of fruits diluted in HBSS to produce a concentration range of 1000–10,000 µg/mL applied at 50 μL/well. Each treatment was applied for 2 h and was compared with cell viability in response to HBSS only. After 2h, all treatments were removed and the cells were washed once with HBSS (74 μL/well). To measure cell viability, 74 µl/well CyQUANT^®^ NF DNA binding dye was added to the cells and the cells were incubated for 60 min at 37 °C before fluorescence was measured using 485 nm excitation and 530 nm emission using a Spectramax M3 multi-mode microplate reader.

### 2.3. Statistical Analysis

Data is presented as the mean percentage (±SEM) of three replicates for each treatment. Cytotoxicity is expressed as the percentage of viable cells remaining after extracts treatment compared to the control. Variable response curves were fitted by GraphPad Prism version 8 (GraphPad Software, San Diego, CA, USA).

## 3. Results and Discussion

**Figure 1:**
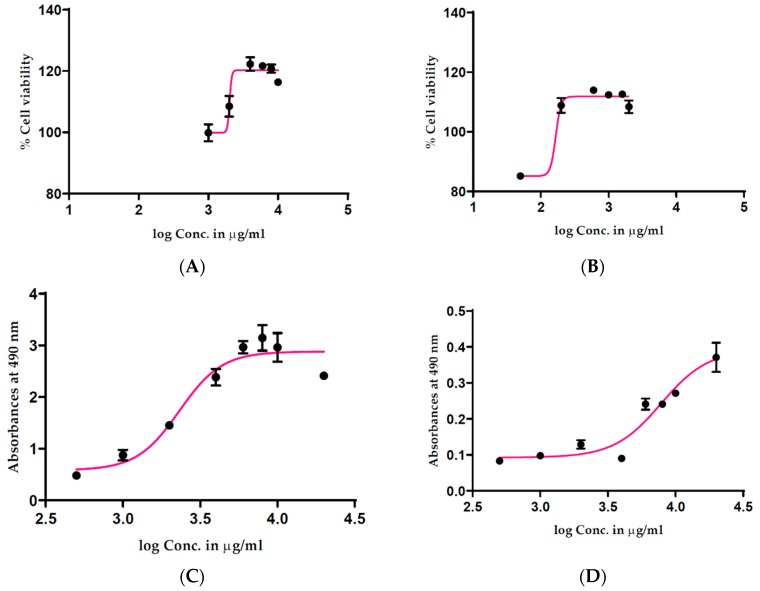
Effect of *T. ferdinandiana* methanolic (**A**) and water (**B**) extracts of fruits on HT29-MTX-E12 cell viability measured using the CellTiter 96^®^ AQ_ueous_ One Solution Cell Proliferation Assay, and in a cell-free system (**C**,**D**), respectively. All concentrations were tested in triplicate with mean values ± SEM used to construct dose–response curves. Conc. denotes concentration.

In this study, we initially employed the CellTiter 96^®^ AQ_ueous_ One Solution Cell Proliferation Assay to measure the effect of antioxidant-rich *T. ferdinandiana* extracts on the viability of human HT29-MTX-E12 cells. Cells were treated with 1000–10,000 µg/mL extracts for 2 h. Cell viability was expressed as a percentage compared to untreated cells with treatment–response curves drawn by plotting the cell viability percentage against the extract treatment concentration. An unexpected increase in cell viability was observed at higher treatment concentrations (Figure 1), suggesting a 20% increase in cell viability following the 2 h treatment in response to 4000–8000 µg/mL of methanolic (Figure 1A) and water extracts (Figure 1B). Conversely, cell viability appeared to be lower with lower treatment concentrations of the extracts. These results suggested interactions between the cell viability assay reagents and the extracts.

**Figure 2:**
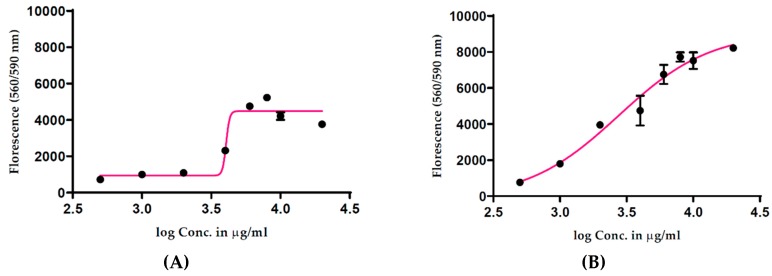
Effect of *T. ferdinandiana* methanolic (**A**) and water (**B**) extracts of fruits in a cell-free system using the CellTiter-Blue^®^ Cell Viability Assay. All concentrations were tested in triplicate with mean values ± SEM used to construct dose–response curves.

A cell-free experiment was then performed to determine whether the fruit extract could directly bio-reduce the cell viability assay reagent, creating false cell viability results. All the absorbance readings from methanolic (Figure 1C) and water (Figure 1D) extracts increased in accordance with an increase in treatment concentrations, providing evidence that even in the absence of cells, the fruit extracts were reducing the assay reagent. In an attempt to identify a more suitable viability assay, we performed a CellTiter-Blue^®^ Cell Viability Assay, commonly known as the resazurin assay. This assay is based on the reduction of resazurin to its highly fluorescent product resorufin by metabolically active cells, with fluorescence proportional to the number of viable cells. Results of a cell-free experiments using the CellTiter-Blue^®^ Cell Viability Assay showed increased fluorescence with increasing concentrations of the methanolic (Figure 2A) and water (Figure 2B) extracts of *T. ferdinandiana* fruits. No autofluorescence was observed in response to the extracts alone.

**Figure 3:**
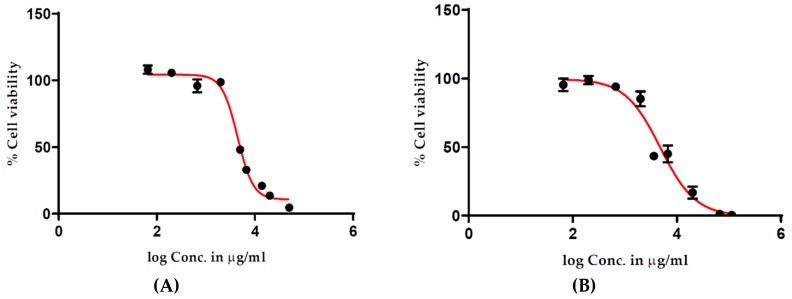
Effect of *T. ferdinandiana* methanolic (**A**) and water (**B**) extracts of fruits on HT29-MTX-E12 cell viability measured using the CyQUANT^®^ NF Assay. All concentrations were tested in triplicate with mean values ± SEM used to construct dose–response curves.

To identify a cell viability assay compatible with antioxidant-rich extracts that could provide a more accurate estimation of cell viability, the CyQUANT^®^ NF Cell Proliferation Assay was employed. The CyQUANT^®^ NF Cell Proliferation assay measures cellular DNA content via fluorescent dye binding, and with cellular DNA content highly regulated and closely proportional to the cell number, cell viability is measured by fluorescence [8]. The CyQUANT^®^ NF assay results are presented in Figure 3 and show a typical treatment–response curve, with reduced cell viability observed at higher concentrations of the methanolic (Figure 3A) and water (Figure 3B) extracts of fruits. Subsequently, by comparing the results from the different assays, it appears as though the antioxidants present in the extract promote the conversion of MTS to formazan, producing false positive viability results.

**Figure 4:**
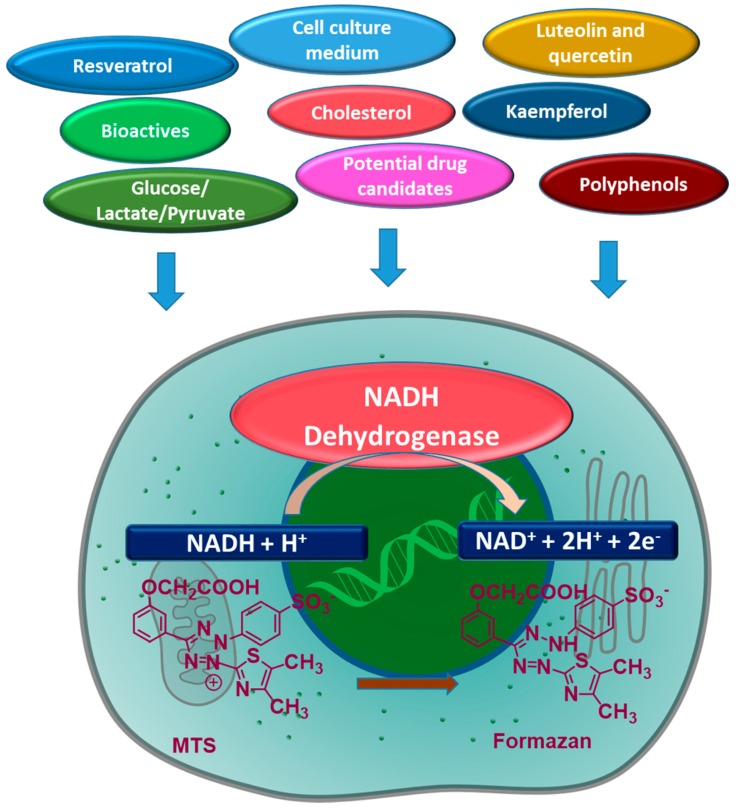
Factors affecting the reduction/conversion of MTS (3-(4, 5-dimethylthiazol-2-yl)-5-(3-carboxymethoxyphenyl)-2-(4-sulfonyl)-2H-tetrazolium) to formazan by metabolically active cells.

*T. ferdinandiana* fruits are naturally rich in antioxidants like ellagic acid and vitamin C, and based on the outcomes of this study, can directly bio-reduce cell viability assays dependent upon the activity of dehydrogenase enzymes. In support of the findings presented here, other studies report the direct reduction of MTS to formazan by phenolic antioxidants (Figure 4), including kaempferol and ellagic acid, in the absence of cells [2,3]. The increased cell viability observed in the CellTiter 96^®^ AQ_ueous_ One Solution Cell Proliferation Assay after treating cells with *T. ferdinandiana* fruit extract can be explained by the presence of redox active antioxidants, such as vitamin C [9]. Other studies involving plant extracts containing vitamin E, luteolin, quercetin, flavones, flavonones, alkaloids, glycosides, and epigallocatechin-3-gallate (Figure 4) have also reported direct interaction with MTT in cell-free systems [2]. The MTT assay, and to a lesser degree MTS, and other related assays like XTT (2,3-bis-(2-methoxy-4-nitro-5-sulfophenyl)-2*H*-tetrazolium-5-carboxanilide) or WST (water soluble tetrazolium salts), are a widely exploited approach for measuring cell viability in response to different targets such as drugs.

The reduction of MTT, however, can occur through many different mechanisms, ultimately impacting cell viability results. For example, intracellular MTT reduction can occur through NADH and NADPH by oxidoreductases or superoxides, whereas extracellular reduction can occur through NADH and NADPH by cell surface oxidoreductases. Mitochondrial and non-mitochondrial enzymes, endoplasmic reticulum, cytosol, and plasma membranes can also participate in reducing MTT [10]. Factors that influence intracellular trafficking of MTT-formazan, such as disturbances in cellular metabolic and energy homeostasis, rate of endo- or exocytosis, oxidative stress, glucose uptake and glycolysis, lactate and pyruvate levels, and formazan, can also impact MTT reduction [10]. Off-target effects of the investigated molecules, such as potential drug candidates and other bioactives, can also cause under or over-estimation of cell viability [2]. Cell culture medium composition, cell growth state, concentration and consumption rate of energy supply metabolites, nanoparticles, polypeptides, and X-ray radiation may also impact MTT reduction and measured cell viability [2].

All cell viability assays offer advantages and limitations; for example, the MTT/MTS assays are easy to perform but variable metabolic behaviour can be observed under different cell culture conditions and the solubility of the insoluble formazan products can sometimes be problematic, whereas WSTs are water soluble and easy one-step assays. However, variable metabolic behaviour under different cell culture conditions can also be observed with WSTs. DNA assays such as CyQUANT^®^ can be directly correlated with proliferation, however, black plates are needed and the assay requires RNase treatment for the detection of DNA and vice versa [11].

In this study, methanolic and water extracts of *T. ferdinandiana* fruits were incubated with MTS (Figure 1A,B) in a cell-free setting, producing direct MTS reduction after a 2-h incubation period, whereas positive and negative controls did not produce formazan. Based on the findings presented here and by others, different variables, such as the pH and osmolarity of cell culture media, positive and negative control substances, interactions with MTS in a cell-free system, and washing steps, are important considerations when performing cell viability assays. The application of the CellTiter 96^®^ AQ_ueous_ One Solution Cell Proliferation Assay as a screening system for plant extracts requires pre-screening in cell-free systems to determine whether the extracts exhibit a reductive capacity that might prevent optimum performance of this assay. Cell washing steps can be added to reduce the potential interference by reductive extracts, however, washing cannot prevent the impact of redox active extracts on intracellular redox status that will also impact the cell viability measured through MTS or MTT.

## 4. Conclusions

The results in the present study demonstrated that the CellTiter 96^®^ AQ_ueous_ One Solution Cell Proliferation Assay underestimated the anti-proliferative effects of *T. ferdinandiana* extracts, highlighting the importance of determining the most appropriate cell viability system for the extracts being investigated. The overestimation of cell viability in response to the antioxidant-rich *T. ferdinandiana* fruit extracts is most likely due to increased dehydrogenase activity in the treated cells as well as the intrinsic reductive potential of the fruit extracts to reduce the CellTiter 96^®^ AQ_ueous_ One Solution Cell Proliferation Assay reagent. To identify more appropriate cell viability assays for antioxidant-rich extracts, the method cannot be based on redox reactions, therefore more appropriate assays should be targeted to cellular protein content, intracellular protein release, or DNA measurement.

## References

[1] Xu M., McCanna D.J., Sivak J.G. (2015). Use of the viability reagent prestoblue in comparison with alamarblue and mtt to assess the viability of human corneal epithelial cells. J. Pharmacol. Toxicol. Methods.

[2] Stepanenko A.A., Dmitrenko V.V. (2015). Pitfalls of the mtt assay: Direct and off-target effects of inhibitors can result in over/underestimation of cell viability. Gene.

[3] Wang P., Henning S.M., Heber D. (2010). Limitations of mtt and mts-based assays for measurement of antiproliferative activity of green tea polyphenols. PLoS ONE.

[4] Konczak I., Zabaras D., Dunstan M., Aguas P. (2010). Antioxidant capacity and hydrophilic phytochemicals in commercially grown native Australian fruits. Food Chem..

[5] Konczak I., Roulle P. (2011). Nutritional properties of commercially grown native Australian fruits: Lipophilic antioxidants and minerals. Food Res. Int..

[6] Williams D.J., Edwards D., Pun S., Chaliha M., Sultanbawa Y. (2014). Profiling ellagic acid content: The importance of form and ascorbic acid levels. Food Res. Int..

[7] Navarro M., Sonni F., Chaliha M., Netzel G., Stanley R., Sultanbawa Y. (2016). Physicochemical assessment and bioactive properties of condensed distillers solubles, a by-product from the sorghum bio-fuel industry. J. Cereal Sci..

[8] Jones L.J., Gray M., Yue S.T., Haugland R.P., Singer V.L. (2001). Sensitive determination of cell number using the cyquant^®^ cell proliferation assay. J. Immunol. Methods.

[9] Bruggisser R., von Daeniken K., Fau-Jundt G., Jundt G., Fau-Schaffner W., Schaffner W., Fau-Tullberg-Reinert H., Tullberg-Reinert H. (2002). Interference of plant extracts, phytoestrogens and antioxidants with the mtt tetrazolium assay. Planta Med..

[10] Berridge M.V., Herst Pm Fau-Tan A.S., Tan A.S. (2005). Tetrazolium dyes as tools in cell biology: New insights into their cellular reduction. Biotechnol. Annu. Rev..

[11] Quent V.M.C., Loessner D., Friis T., Reichert J.C., Hutmacher D.W. (2010). Discrepancies between metabolic activity and DNA content as tool to assess cell proliferation in cancer research. J. Cell. Mol. Med..

